# Treatment of rabbit growth plate injuries with oriented ECM scaffold and autologous BMSCs

**DOI:** 10.1038/srep44140

**Published:** 2017-03-07

**Authors:** Wenchao Li, Ruijiang Xu, Jiangxiang Huang, Xing Bao, Bin Zhao

**Affiliations:** 1 Department of Pediatric Surgery, Chinese People’s Liberation Army General Hospital, Beijing, 100853, China; 2 Department of Orthopedics, Chinese People’s Liberation Army General Hospital, Beijing, 100853, China

## Abstract

Tissue-engineered technology has provided a promising method for the repair of growth plate injuries using biocompatible and biodegradable scaffolds and appropriate cells. The aim of this study was to fabricate oriented ECM scaffolds to imitate the material and structure of a natural growth plate and to investigate whether BMSCs in a scaffold could prevent the formation of bone bridges in an injured growth plate. We developed a natural, acellular and oriented scaffold derived from a growth plate. The oriented scaffold was fabricated using new freeze-drying technology and by cross-linking the microfilaments in the growth plate. From histological examination, the scaffold contained most of the ECM components including GAG and collagen II without cell DNA fragments, and SEM revealed that oriented scaffold had a uniform aperture in the transverse plane and columnar structure in length plane. Cytotoxicity testing with MTT showed no cytotoxic effect of the scaffold extracts on BMSCs. Autogenous BMSCs in oriented scaffolds promoted the regeneration of neogenetic growth plate when repairing an injured growth plate and prevent the formation of bone bridges to reduce the angular deformity and length discrepancy in the proximal tibia in rabbits. The well-characterized ECM-derived oriented growth plate scaffold shows potential for the repair of injured growth plates in young rabbits.

Growth plates are highly susceptible to damage and have limited intrinsic regeneration and self-repair capacity as result of the formation of bone bridges. Bone bridges in an injured growth plate may result in length discrepancies and angular deformities of limbs, which present a challenging problem. In the present study, tissue engineering is demonstrated to be a promising alternative therapy through the use of biocompatible and biodegradable scaffolds, appropriate cells and environmental factors in repairing a growth plate injury. The scaffold, which is a major component of tissue engineering, can provide a living microenvironment in which cells can be seeded to promote adhesion, aggregation and proliferation. A broad range of scaffolds including chitin^1^, atelocollagen gel^2^, agarose^3^, demineralized bone matrix^4^ and alginate-polylysine-alginate^5^ have used to treat injured growth plates with varying success.

Growth plate extracellular matrix (ECM) is a complex mixture of structural and functional proteins, glycoproteins, and proteoglycans arranged in a unique, tissue-specific three-dimensional ultrastructure. Natural ECM scaffolds can provide an environment that resembles the extracellular surroundings and that contains informational signals when seeding cells, which may be functionally superior to synthetic polymers. Compared with polyglycolic acid (PGA) scaffolds, ECM was more efficient at producing high-quality hyaline cartilage-like tissues using rabbit chondrocytes both *in vitro* and *in vivo*^6^. This ECM scaffold was composed of matrix molecules from cartilage including sulfated glycosaminoglycans (GAGs) and type II collagen^7^,^8^. ECM not only plays a major role in maintaining the function of tissue but also interacts and communicates directly with the seeded cells via cell receptor/matrix interactions. ECM scaffolds can provide attachment sites for cell surface receptors and a reservoir for signaling factors that modulate such diverse host processes as angiogenesis, vasculogenesis, cell migration, cell proliferation and orientation, inflammation, immune response and wound healing^9^.

Additionally, optimal scaffolds require a three-dimensional structure and a high porosity with interconnection, balanced biodegradability and biocompatibility. Our previous study demonstrated that cartilage ECM-derived 3-D porous acellular matrix scaffold could provide a suitable 3-D environment to support the adhesion, proliferation and differentiation of bone marrow-derived mesenchymal stem cells (BMSCs)^10^,^11^. However, histological results showed that neogenetic chondrocytes were irregularly scattered in the cartilage defect lacking the normal biological function of the natural growth plate. The growth plate has columns of chondrocytes in three principal layers: resting, proliferative, and hypertrophic zones. The combination of chondrocyte proliferation, enlargement of maturing chondrocytes in the hypertrophic zone, and the ECM play a major role in vertical bone growth. Vertical and transverse septae, which maintain the chondrocytes in a column-wise orientation, are resorbed by chondroclasts or osteoclasts from the underlying primary spongiosum^12^,^13^. The characteristic structure of the growth plate is the basis of bone growth. The designed structure of the scaffolds should mimic the native cartilage of the growth plate, which has an oriented structure of chondrocytes associated with its mechanical and biological functions. The oriented architecture of the scaffold should possess superior biomechanical properties and protect cells from early critical compression prior to the secretion of abundant ECM^14^. Therefore, scaffolding with a biomimetic-oriented architecture is the major objective in tissue-engineered growth plate cartilage in our experiment.

The purpose of this study was to (1) fabricate oriented growth plate ECM-derived scaffolds to imitate the material and structure of the nature growth plate, (2) assess the characteristic and compatibility of oriented ECM scaffolds and (3) investigate whether antogeneic BMSCs within oriented ECM scaffolds could prevent the formation of bone bridges and promote the regeneration of neogenetic chondrocytes in the treatment of injured growth plate (Fig. 1).

## Results

### Characterization of growth plate cartilage microfilament

**Figure 2:**
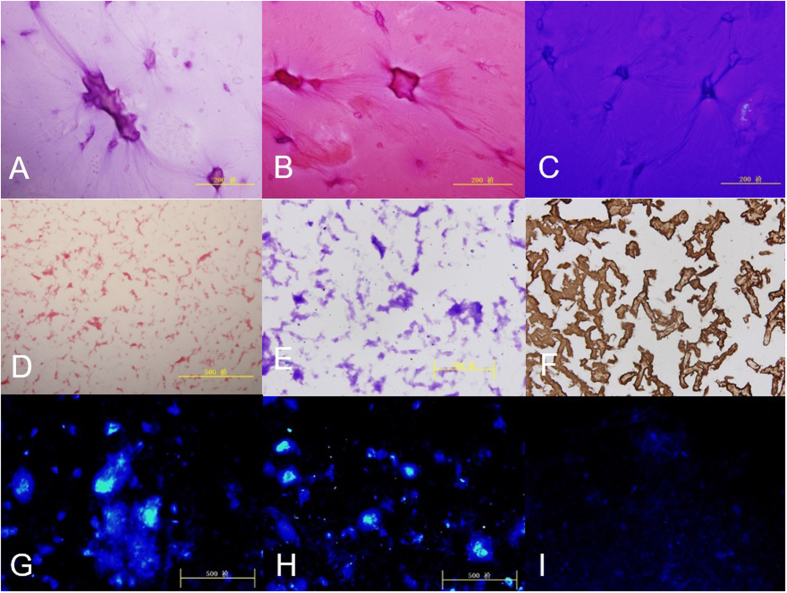
(**A**) HE staining showed that suspension of ECM microfilament was ivory and ropiness; (**B** and **C**) The positive of staining showed fibriform of microfilament was disorderly mixed with cell debris; (**D** and **E**) The positive of Safranin O and TB staining showed purified microfilaments of growth plate richly contained GAG. (**F**) The positive staining showed ECM microfilaments contained collagen II. (**G** and **H**) Hoechst 33258 staining showed cell debris of growth plate in suspension before centrifuged with different velocity; (**I**) No cells or cell fragments were present in scaffold after different velocity of centrifugalization.

After pulverization in a moistened environment, the ECM microfilament suspension was ivory and ropy (Fig. 2A). The macroscopic view showed that the fibriform of the microfilaments were crude with cell debris, and there was a disorderly arrangement (Fig. 2B and C). After centrifuging at a different velocity, the cell debris in the suspension was removed with the surplus of purified growth plate microfilaments (Fig. 2D). Positive histochemical staining showed that the ECM microfilaments contained GAG and collagen II (Fig. 2E and F). Before centrifugation, Hoechst 33258 staining showed that the majority of the DNA debris remained in the suspension (Fig. 2G and H). The staining showed that no cells or cell fragments were present in the scaffold after centrifugation at different velocities (Fig. 2I).

**Figure 3:**
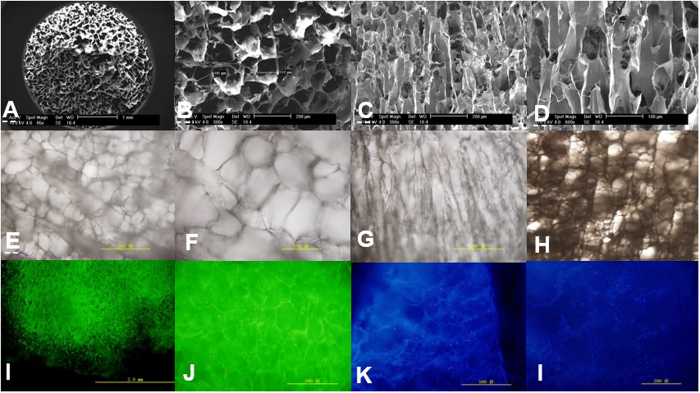
(**A**) SEM micrograph (low power) of cartilage ECM-derived porous scaffold; (**B**) SEM micrograph of transverse plane (high power) of porosity structure with regular appearence; (**C** and **D**) SEM micrograph of length plane (high power) of column wise orientation; (**E** and **F**) Inverted microscope showed transverse plane of interconnected porous and spongelik; (**G** and **H**) Inverted microscope showed length plane of columnwise orientation; (**I** and **J**) The staining of AO/PI showed hondrocytes in scaffold grow well with little dead cells; (**K** and **I**) The staining of Hoechst 33258 showed DNA of cell nucleus in ECM scaffold.

After decellularization of microfilaments, the frozen suspension and crystallization of the orientation were lyophilized to prepare the oriented ECM scaffold. Scanning electron microscopy (SEM) showed a porous structure in the transverse plane (Fig. 3A and B) and a column-wise orientation in the length plane (Fig. 3C and D). Inverted microscopy showed a transverse plane of interconnected porous and a sponge-like structure (Fig. 3E and F) and the length-wise plane had a column-wise orientation (Fig. 3G and H). Scaffolds had a bulk density of 0.073 ± 0.0096 and a porosity of 91.7 ± 2.93%, as determined by mercury intrusion porosimetry. The SEM results showed that the pore diameter was 114.1 ± 8.08 μm. Positive Safranin O and TB staining showed that the ECM scaffold retained GAGs after decellularization. Immunohistochemical staining with collagen II antibodies revealed collagen II components in the ECM scaffold. Cytotoxicity testing using MTT revealed no significant difference in the absorbance among different extracts, which shows no cytotoxic effect of the scaffold on BMSCs.

### Cell attachment and viability of the cell-scaffold constructs

After BMSCs were added to the ECM scaffold, inverted microscopy showed that the cells adhered to the surface of the scaffold, proliferated and fused according to the columnar lacuna of the scaffold. The majority of cells displayed a round or elliptical morphology similar to chondrocyte-like cells distributed according to the scaffold. High magnification revealed moderate cell adhesion onto the scaffolds with ECM and collagen fibers around the cells. Live cells adhered to the rim of the pores or distributed inside the scaffolds. The cell viability assessments using fluorescence microscopy was performed. AO/PI staining showed bright green cell nuclei in the scaffolds with a small numbers of red nuclei in scaffold (Fig. 3I and J). Confocal microscopy of the cell-scaffold constructs using Hoechst 33258 staining revealed cells with blue fluorescence after 48 h of culture (Fig. 3K and L).

### Histological findings

**Figure 4:**
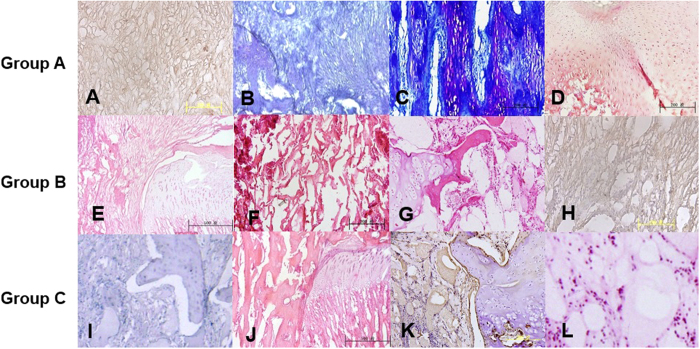
In group A: (**A**) at 4 weeks, positive immunohistochemistry staining of collagen II showed ECM scaffolds contained collagen II around BMSCs with minor degradation. (**B**) at 8 weeks, the positive TB staining showed BMSC gradually differentiate to chondrocytes and secrete GAG around ECM. Scaffold and normal growth plate fused well without inflammatory reactions; (**C**) neogenetic chondrocytes in ECM scaffold were arranged according to column structure; (**D**) at 16 weeks, growth plate defect was full of column chondrocytes; In group B: (**E**) at 4 weeks, scaffold was gradually degradation with major residuals; (**F**) The positive staining of collagen II showed ECM scaffolds was rich of collagen II in growth plate defect; (**G**) at 8 weeks, ECM scaffold was significant degradation with bone trabeculas in growth plate defect; (**H**) at 16 weeks, the interior of ECM scaffold was full of fibrous tissues and bone trabeculas; (**I**) In group C, at 8 weeks, bone trabeculas and fibrous tissues was full of growth plate defect; (**J**) the injured growth plate turns to closure.

In group A, at 4 weeks, the positive immunohistochemistry staining for collagen II showed ECM scaffolds containing collagen II around the BMSCs with minor degradation (Fig. 4A). At 8 weeks, positive TB staining showed that the BMSCs gradually differentiated into chondrocytes and secreted GAG around the ECM. The scaffold and the normal growth plate fused well without inflammatory reactions (Fig. 4B). Neogenetic cells were arranged according to the ECM scaffold in a column structure (Fig. 4C). At 16 weeks, the growth plate defect in the tibia was filled with neogenetic growth plate, which was similar to cells in the resting zone with considerable number of cells having small volumes (Fig. 4D). Histochemical staining showed that the neogenetic chondrocytes could produce ECM including GAG and collagen II. At 20 weeks, the normal growth plate has a tendency to close, and the repaired growth plate demonstrated columns of chondrocytes as is the case with the extracellular matrix.

In group B, at 4 weeks, the ECM scaffold gradually degraded with major residuals (Fig. 4E and F). At 8 weeks, the ECM scaffold was residual and bone trabeculae were around the scaffold fragments (Fig. 4G). At 16 weeks, the injured growth plate began to close, and bone trabeculae and fibrous tissues were growing in the plate defect (Fig. 4H). In group C, at 4 weeks, bone trabeculae and fibrous tissues filled the growth plate defect. At 8 weeks, the injured growth plate changed into bone bridges (Fig. 4I). At 16 weeks, the normal and injured growth plate began to close (Fig. 4J).

### Radiologic studies

#### Angular deformity

**Figure 5:**
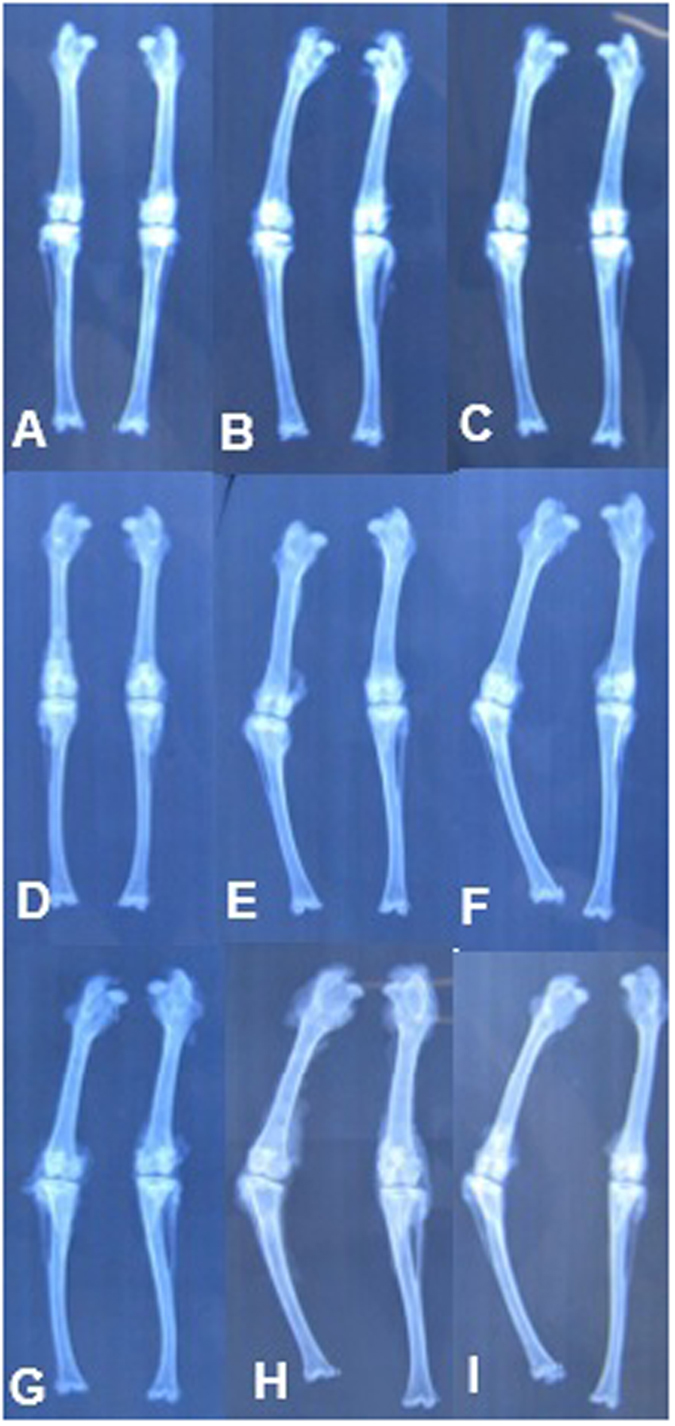
In group A: (**A**) at 4 weeks; (**B**) at 8 weeks and (**C**) at 16 weeks. In group B: (**D**) at 4 weeks; (**E**) at 8 weeks and (**F**) at 16 weeks. In group C: (**G**) at 4 weeks; (**H**) at 8 weeks and (**I**) at 16 weeks.

At 4 weeks after surgery, angular deformities were present in the tibias of all groups. The control group had a greater increase in the angular deformity than group A and B. However, there was no significant differences between group A and group B. At 8 weeks, the angular deformity in group B was gradually increased to be larger than the angular deformity of group A. At 16 weeks, the angular deformities of group B and C were greater than that in group A. However, there was no significant difference between group B and group C. At 20 weeks, the angular deformity in all groups had a tendency to be stable at 15.9 ± 3.83, 27.1 ± 5.23 and 32.1 ± 2.85°, respectively. Group A showed the smallest angular deformity among all groups (p < 0.001). *Length discrepancy*: At 4 weeks after surgery, a length discrepancy was present in all tibias from all groups. The discrepancy in Group A and B was less than the discrepancy in the control group from 4 to 8 weeks after surgery. There was no significant difference between group A and group B. At 12 weeks, group B showed a tendency to have a length discrepancy, and this discrepancy was greater than the discrepancy in group A. At 16 weeks, the length discrepancies from group B and group C gradually increased during this period. At 20 weeks, there was a significant difference between group A and group B and C (Fig. 5) (Table 1).

## Discussion

In our experimental study, we successfully developed a novel growth plate made of an ECM-derived oriented acellular scaffold to imitate the column structure of natural growth plate, and we evaluated and characterized and biocompatibility of this ECM scaffold. BMSCs successfully adhered and distributed in the oriented scaffold in a manner similar to the column structure of natural chondrocytes. Furthermore, implantation of the oriented ECM scaffold with autogeneic BMSCs in a growth plate defect in juvenile rabbits generated superior functional tissue-engineered cartilage, and radiologic results showed that the angular deformity and length discrepancy of the tibia in experimental group was significantly less than in the ECM groups and the blank group (P < 0.005).

The concepts guiding the development of tissue-engineered cartilage rest on the selected biomaterial or the newly designed scaffold and on optimal cell seeding. Scaffolds provide the living environment for cells that are seeded and are an important factor in tissue-engineered growth plates. The ECM in the growth plate is a complex network composed of collagen, fibronectin and other proteins all of which are interlaced with proteoglycans^15^. Natural scaffolds with ECM components and structures are of particular interest as they can promote structurally and functionally constructive tissue-engineered remodeling. ECM scaffolds have high tissue compatibility and have the potential to retain cytokines, growth factors, and other functional proteins. ECM scaffolds can provide not only structural guidance for cell growth and tissue morphogenesis but can also play a functional role, such as is enhancing cell attachment and metabolism^7^. We selected the ECM of growth plate cartilage as the material for the scaffold to preserve the main constituents of the native cartilage including GAG and type II collagen, to possess good biocompatibility and to provide a natural microenvironment for the support of BMSC attachment, proliferation and differentiation into chondrocytes^7^.

Our previous studies showed ECM-derived porous scaffold as a promising biomaterial for cartilage tissue engineering to be used to repair cartilage defect with successful results. However, cells seeded in the scaffold within an injured growth plate showed that neogenetic chondrocytes were irregularly scattered within the cartilage defect. The irregular arrangement of chondrocytes could not play a normal function with respect to bone growth. The suitable structure of the scaffolds should imitate a natural growth plate, which has oriented column of chondrocytes along with its mechanical structure and physiological functions^14^. Growth plates are highly organized cartilage structures entrapped between the epiphyseal and metaphyseal bone at the distal ends of long bone. The length-wise growth of long bone is referred to as endochondral ossification in which the growth plate is replaced by neogenetic bone in a coordinated fashion. The growth plate can be divided into horizontal zones of chondrocytes at different stages of differentiation^16^. The growth plate has a column structure of chondrocytes that includes three principal layers: the resting, proliferative, and hypertrophic zones. Chondrocytes in the resting zone are irregularly scattered in a bed of cartilage matrix, whereas cells in the proliferative and hypertrophic zones are arranged in columns parallel to the long axis of the bone^17^. The structure of the columns and layers of chondrocytes is the basis of vertical bone growth. This spatial orientation directs growth in a specific direction and is, thus, responsible for the elongated shape assumed by endochondral bones. ECM scaffolds including GAG and collagen II without cell DNA fragments process excellent biocompatibility without cytotoxic effect of the scaffold extracts on BMSCs. Besides, oriented ECM scaffold revealed the uniform aperture at transverse plane and columnar structure at length plane, which imitate the structure of natural growth plate.

In this study, we developed a novel method of tissue decellularization combining physical pulverization in which the growth plate was processed to produce cartilage microfilament suspensions. The novel decellularization method was the modification of a protocol previously used for porcine bladder matrix. In short, we used physical pulverization and osmotic lysis of the cells using a non-ionic detergent (TritonX-100) to solubilize the cell fragments, protease inhibitors (PMSF and EDTA) to inhibit autolysis, and nucleases (DNase and RNase) to digest nuclear materials along with extensive washing steps. Finally, we fabricated the oriented growth plate scaffold by using an improved freeze-drying technique. Due to the existence of hypothermia during phase separation, frozen crystals were observes oriented along the temperature gradient, and this resulted in vertical microtubules following the solvent removal by freeze-drying^18^.

The column oriented ECM scaffold had a parallel arrangement of microtubules, an appropriate pore size and a high porosity, which makes the scaffold suitable for BMSCs proliferation and differentiation^19^. Scaffolds with a biomimetic-oriented architecture are an important requirement for a tissue-engineered growth plate. The compressive modulus of the oriented scaffolds was attributed to the microtubule walls and offered increased capacity to support more compressive stress and provide effective support to the growth plate defect. Planka *et al*.^20^ reported the scaffold with collagen containing chitosan nanofibers in repair of growth plate injury, and it were not stable enough and need to be made of a three-dimensional scaffold with a self-supporting structure of collagen fibres. Besides, Jin *et al*.^4^ used demineralized bone matrix (DBM) scaffold with chondrocytes in treatment of growth plate defect, without the columnar arrangement of chondrocytes and revealing that DBM scaffold can only prevent the bone bridge forming in a short time.

In this study, histological and immunohistochemical examination of the scaffolds showed that oriented microtubules induced BMSC migration into the core of the scaffold, and the structure may have caused an enhancement of the mechanical properties to protect the cells from early critical compression before sufficient ECM was deposited. Additionally, the scaffolds had a bulk density of 0.073 ± 0.0096 and porosity of 91.7 ± 2.93% as determined by mercury intrusion porosimetry. The SEM results showed that the pore diameter was 114.1 ± 8.08 μm. These parameters of the oriented scaffold were suitable for cells adhesion and proliferation and help protect cells from early critical compression prior to the secretion of abundant ECM. *In vitro*, BMSCs could adhere and align in the direction of the vertical microtubules and proliferate within ECM scaffold, which thus imitated the physiological structure of native growth plate. The oriented structure facilitates the transport of culture medium and the exchange of metabolites to accelerate the proliferation of cells according to the gap of the scaffold. Scaffolds could possess superior biomechanical properties and protect cells from early critical compression. Furthermore, oriented scaffolds fabricated from various materials using diverse techniques have been shown to possess superior strength and compressive modulus compared with typical non-oriented scaffolds in the dry state *in vitro*^21^.

The source of seeded cells is an additional factor critical to the reconstruction of the injured growth plate. Autologous chondrocytes are thought to be the optimal cells for targeted regeneration of cartilage. However, the supply of chondrocytes is very limited, and excising these cells may cause additional injury. MSCs harvested by bone marrow biopsy have been reported as an alternative cell source due to their characteristic capacity for rapid proliferation and multiple differentiated potential^22^. BMSCs display multi-lineage differentiation potential for osteoblasts, chondrocytes, and adipocytes^23^. Planka *et al*. reported that preventive transplantation of MSCs prevented the development of angular deformity and eliminated growth arrest in femur physeal injuries in pigs^24^. Chen *et al*. also used cultured MSC derived from periosteum and agarose in large (50%) defects at the proximal tibial physis, and growth arrest with angular deformation and loss of length of the tibia was corrected^3^. Azarpira *et al*. reported that growth arrest model in which the lateral 50% of the distal femoral physis was removed and was filled with a MSC-based chitosan scaffold with a resulting trend toward less angular deformity^25^. The aspects of autogenous vs. allogeneic MSC transplantation into the injured physeal growth zone of extremity bones remain a problem. We selected isolated autogenous BMSCs as the cells that were seeded into scaffolds to reduce possible influencing factors. Autogenous BMSCs from iliac bone do not cause secondary damage as would be the case with chondrocytes isolated from the epiphyseal plate or articular cartilage.

In this study, the efficacy of autogenic BMSCs combined with oriented an ECM scaffold was assessed for treatment of an injured growth plate and in its ability to reduce abnormalities in rabbit tibias. In group A, at 8 weeks, positive TB staining showed that the BMSC gradually differentiated to chondrocytes and secreted GAG around the extracellular matrix. The scaffold and the normal growth plate fused well without an inflammatory reaction. Neogenetic cells were arranged in the ECM scaffold in a column-like structure. At 16 weeks, the tibia growth plate defect was filled with neogenetic growth plate, which had a considerable number of small-volume cells similar to the resting zone. Immunohistochemical staining showed that the neogenetic chondrocytes could produce extracellular matrix including glycosaminoglycan and collagen II. BMSCs in the oriented scaffold gradually differentiated into chondrocytes as the ECM scaffold degraded. The cells were arranged in columns in the ECM scaffold, which is similar to arrangement of natural chondrocytes. The arrangement of cells improved the proliferation and differentiation of chondrocytes into the typical columns observed in the growth plate. Histological examination showed that the neogenetic chondrocytes replicated at a slow rate and acted as the stem-like cells that replenished the pool of proliferative chondrocytes. Subsequently, the cells replicated at a high rate and the resulting daughter cells lined up along the long axis of the bone. As a result, clones of the chondrocytes were arranged in columns parallel to this axis, which is a process critical to the formation of bones with an elongated shape.

The angular deformity and length discrepancy of the rabbit tibias were analyzed to evaluate the growth function of the neogenetic growth plate. At 4 weeks, the angular deformity and length discrepancy of tibias were significant greater than those in group A and group B. In the early period, the ECM and ECM scaffold could fill the growth plate defect to prevent the formation of bone bridges. With the degradation of the ECM, bone and fibrous tissue then grew into the defects and the function of ECM gradually declined. At 8 weeks, the angular deformity and length discrepancy in group B were significantly greater than group A but less than group C. From 12 weeks to 20 weeks, there was no significant difference between group B and group C (P < 0.05). In group A, BMSCs in the ECM scaffold gradually caused the growth plate to recover, and there was long bone growth. Therefore, the deformities in group A were always less than in others groups from 8 weeks to the end of the experiment.

Although the results of the experiment were encouraging and oriented ECM processes excellent biocompatibility, the experiments show that BMSCs in the tissue-engineered scaffold composite can only efficiently prevent bone bridge formation in acutely injured rabbit models. However, patients with growth plate injuries typically cannot be diagnosed and treated in time. Patients with limb deformities typically suffer from growth plate injury over a long period of time. Clinically, the shape of scaffold should be fabricated according to the defect in the growth plate. Bone bridges within the growth plate were measured using three-dimensional CT scan. Additionally, the microenvironment of the injured growth plate was considered to play an important role in the formation of bone bridges and the proliferation and differentiation of the BMSCs. Several cytokines, such as transforming growth factor, insulin growth factor I, thyroid hormone and Indian hedgehog, were involved in the proliferation and differentiation of the growth plate chondrocytes. Gene-enhanced tissue engineering may exert a more important function in treating growth plate injuries using these cytokines.

## Conclusion

In this study, we successfully fabricated an oriented ECM-derived growth plate scaffold to imitate the material and structure of the natural growth plate. The ECM scaffolds, which included GAG and collagen II without cell DNA fragments, process excellent biocompatibility, and the scaffold extracts had no cytotoxic effects on BMSCs. SEM revealed that the oriented scaffold had a uniform aperture in transverse plane and a columnar structure in the length plane. Cytotoxicity testing using MTT showed no cytotoxic effect of the scaffold extracts on BMSCs. Finally, BMSCs in the scaffolds promoted the regeneration of neogenetic chondrocytes during the repair of an injured growth plate and prevented the formation of bone bridges, which resulted in reduced angular deformities and length discrepancies in rabbits. Therefore, although the complete prevention of angular and shortening deformities was not possible, the ECM-derived oriented growth plate scaffold shows potential for the repair of injured growth plates in young rabbits.

## Methods

### Preparation of the ECM-derived growth plate scaffold

#### Pulverization and decellularization of the growth plate

All experimental protocols were in compliance with Animal Welfare Act and were approved by the Institutional Animal Care and Use Committee of the Laboratory Animal Research Centre at Chinese PLA General Hospital. The growth plate slices were cut from juvenile New Zealand White rabbits within 2 hours after death using an excess mixture of xylazine (0.1 mg/kg b.w.) and hetamine hydrochloride (10 mg/kg b.w.) in a sterile environment. Slices were washed and shattered into 1 mm^3^ pieces in phosphate-buffered saline (PBS) containing 3.5% (Weight/ Volume, w/v) phenylmethyl sulfonylfluoride (Merck, Darmstadt, Germany) and 0.1% (w/v) EDTA (Sigma, Poole, UK) to inhibit protease activity. The growth plate suspension was centrifuged for 5 min at 2000 rpm, and then suspension was collected and recentrifuged for 5 min at 7000 rpm. Growth plate microfilaments with diameters of approximately 500 nm to 5 mm were prepared after pulverization and differential centrifugation, were then incubated in 1% TritonX-100 in hypotonic Tris-HCl with gentle agitation for 12 h at 4 °C and were then incubated for 12 h in 50 U/mL deoxyribonuclease I and 1 U/mL ribonuclease A in 10 mM Tris-HCl at a pH of 7.5 with agitation at 37 °C after being washed in PBS without protease inhibition. The decellurized cartilage matrix microfilaments were then washed intensively with sterile PBS and made into a 3% (w/v) suspension.

#### Fabrication of growth plate ECM-derived scaffold and cross-linking

Growth plate matrix microfilaments were poured into a cylindrical mold, were frozen at −20 C and −80 C for 1 h, and then lyophilized for 48 h in a freeze dryer (Boyikang, Beijing). The prepared scaffolds were sterilized and cross-linked for 24 h using dehydrothermal. The ECM scaffolds underwent crystallization to create an oriented scaffold with columnar lacuna. Further cross-linking was achieved using water-soluble carbodiimide. ECM scaffolds were immersed in a carbodiimide solution (14 Mm 1-ethyl-3-(3-dimethylaminopropyl) carbodiimide hydrochloride [EDAC] and 5.5 mM N-hydroxysucinimide [NHS]; Sigma) for 2 h at 4 °C. Excess EDAC was rinsed from the scaffolds using PBS. Cartilage ECM-derived scaffolds of approximately 5 mm in diameter and 2 mm in thickness were produced. Finally, the scaffolds were sterilized using 60 Coγirradiation (at 5 mrad).

### Characterization of growth plate ECM scaffolds

#### Microstructure

The ECM scaffolds were cut into slices with a sharp blade. The interior cross sections and vertical sections were consecutively fixed using glutaric acid and osmic acid for 2 h and were dehydrated using a gradient of alcohol. Then, the samples were coated with gold-palladium and examined under scanning electron microscope to investigate the microstructures of the sections (SEM; Hitachi S-520, Japan).

The porosity was measured using the ethanol intrusion method^10^. The volume of the absolute alcohol in a test tube was recorded as V_1._ The scaffold was immersed in the test tube, and the volume of the absolute alcohol was recorded as V_2_. After scaffold was taken out of the absolute alcohol, remaining alcohol was recorded as V_3_. The porosity of the scaffold was, thus, E = (V_1_ - V_3_)/(V_2_ - V_3_). The scaffold was measured five times and the mean value is reported.

To measure the hydrating expansivity of the scaffold, a scaffold was completely immersed in deionized water for 10 min, and the weight was recorded as W. After the scaffold was taken out of deionized and dried in a vacuum drying oven at 50 °C, the scaffold weight was recorded as W_0_. The scaffold was measured five times and the mean value is reported.

#### Histochemistry and fluorescent staining

After pulverization in a moist environment, ECM microfilaments with different velocities of centrifugation were, respectively, stained using Safranin O and toluidine blue (TB) to evaluate the residuals of the ECM. DNA was stained using AO/PI (acridine orange-propidium iodide) and Hoechst dye (bis-benzimide Hochest 33258 pentahydrate; Molecular Probes, Eugene, OR).

The ECM scaffold specimens were fixed in 10% (v/v) neutral buffered formalin. The specimens were dehydrated and embedded in paraffin. Additionally, microfilaments were fixed in 75% (v/v) alcohol and prepared into frozen sections. Specimens were stained with hematoxylin and eosin (HE), Safranin O, TB and collagen II immunohistochemical staining. The residual DNA from the cell nucleus was stained using AO/PI and Hoechst 33258.

### *In vitro* cell culture studies

#### Isolation and culture of BMSCs

New Zealand White rabbits were anesthetized using intramuscular injection of a mixture of xylazine (0.1 mg/kg b.w.) and hetamine hydrochloride (10 mg/kg b.w.). The skin around the ilium was shaved and prepared with povidone-iodine. Bone marrows was then isolated from the iliac crest of the rabbit using a bone marrow needle, and 4~5 mL of bone marrow was fastened to a syringe containing 0.1 mL heparin (3000 units/mL). The nucleated cells from the bone marrow were isolated with Percoll (Amshan-Pharmacia) using gradient centrifugation at a density of 1.073 g/ml and resuspended in complete culture medium composed of Dulbecco’s modified Eagle’s medium (DMEM) (Gibco, USA), 10% fetal bovine serum (FBS), 100 U/mL penicillin, 100 mg/mL streptomycin and 2 mM L-glutamine (GIBCO). The resuspended cells (1 × 10^5^ cells/ml) were plated in 25 cm^2^ culture flasks. The cells were cultured in a humidified atmosphere of 5% carbon dioxide and 95% air at 37 °C, and the medium was replaced with fresh medium every 2 days. Cells before passage 3 were used for the subsequent experiments^26^.

#### Cytotoxicity assay of the ECM scaffolds

The cell viability was measured using the 3-[4,5-dimethyl(thiazol-2yl)- 3,5diphenyl] tetrazolium bromide (MTT, Sigma) method to determine the cytotoxicity of the residual reagents within the scaffold. Then, 3 × 10^3^ BMSCs in 200-μl suspensions were seeded into 96-well plates. After incubation with different concentrations (25%, 50%, 100%) of ECM extract and control medium, on days 1–6, 20 μl of the MTT (1 mg/mL) solution in fresh culture medium was added to each well. After 4 h of additional incubation at 37 °C, the reaction solution was removed and 150 μl of DMSO was added to ensure solubilization of crystals. The optical density values were determined using a microplate reader (Beckman, Fullerton, CA) at 570 nm. Five replicates were performed per sample. Extracts of all scaffolds were prepared as described^27^.

#### Cell attachment and Viability assessment

After the ECM scaffolds were sterilized using 60 Coγirradiation, BMSCs at 1 × 10^7^ cells/ml were seeded onto the scaffolds and allowed to attach for 2 h at 37 °C. Then, scaffolds containing BMSCs were immersed in the DMEM medium. The compounds were incubated *in vitro* at 37 °C in 5% CO_2_. The attachment and viability of the cells in scaffold was evaluated using scanning electron microscopy (SEM).

### Animal model of growth plate injury and transplantation of BMSCs and ECM scaffold

**Figure 6:**
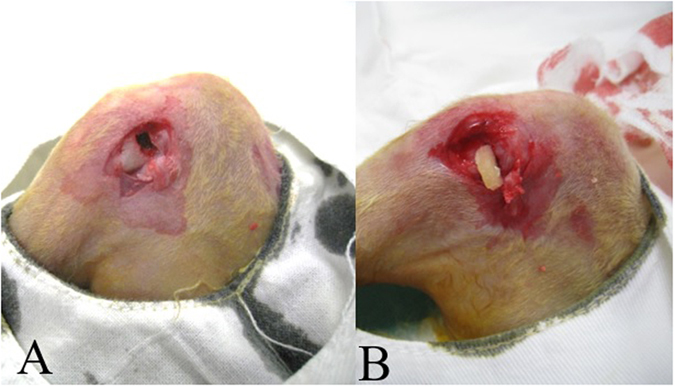
(**A**) After bone periosteum was then incised and stripped, lateral half of growth plate was exposed and carefully removed by scalpel. (**B**) Transplantation of cartilage and BMSCs into growth plate defect.

Seventy-five male immature New Zealand White rabbits (1.2~1.5 kg) aged 8 weeks were used in the experiment. The animals were randomly divided into 4 groups. After the rabbits were anesthetized, the right tibias were shaved and prepared with povidone-iodine. An anteroexternal incision was made over the proximal tibia. After the bone periosteum was incised and stripped, the lateral half of the growth plate was exposed and carefully removed using a scalpel (Fig. 6).

In group A, a compound with the ECM scaffold and the autogenous BMSCs was transferred into the growth plate defect. The periosteum, subcutaneous tissue, and skin were closed in layers. In group B, the ECM scaffolds were transferred into physeal defect. Group C served as a control group, and the periosteum and the skin were sutured immediately after excision of the lateral femur epiphysis without any grafts. All animals were allowed to move freely.

**Figure 7:**
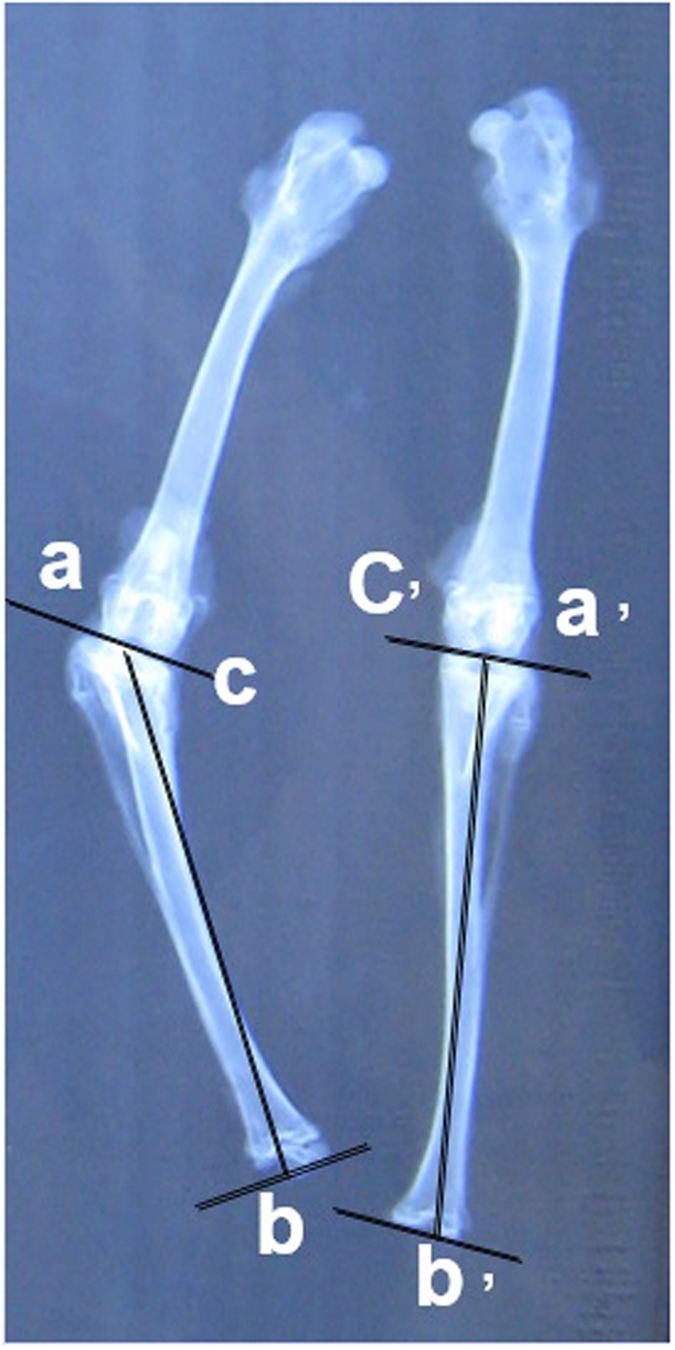
The angular deformity of tibias (right angle - left angle) between the groups was evaluated and recorded. The difference of angular deformity = ∠c’a’b’ - ∠cab; The lengths of tibias were measured and the difference (left tibia length - right tibia length) was calculated and recorded. The difference of lengths = a’b’ - ab.

Rabbits from each group were euthanized at 4-, 8-, 12-, 16,- and 20-week time points. After both of limbs were harvested, anteroposterior radiographs were preformed to evaluate the growth function. The angular deformity of the tibias (right angle − left angle) in all groups was evaluated and recorded. Additionally, the lengths of the tibias were measured and the difference (left tibia length − right tibia length) was calculated and recorded (Fig. 7). Then, the specimens were fixed in 4% paraformaldehyde (pH 7.5) for 7 days and decalcified in 20% EDTA for 6 weeks. The specimens were then embedded in paraffin and cut into 5-μm sections. The sections were stained with standard hematoxylin and eosin (HE) for histologic assessment.

### Statistics

Statistical analysis was performed using SPSS 13.0 software (SPSS Inc; Chicago, IL). The AVONA was used to analyze the radiographic data of the groups at each time point. P < 0.05 was considered statistically significant.

## Additional Information

**How to cite this article**: Li, W. *et al*. Treatment of rabbit growth plate injuries with oriented ECM scaffold and autologous BMSCs. *Sci. Rep.* **7**, 44140; doi: 10.1038/srep44140 (2017).

**Publisher's note:** Springer Nature remains neutral with regard to jurisdictional claims in published maps and institutional affiliations.

## Figures and Tables

**Figure 1:**
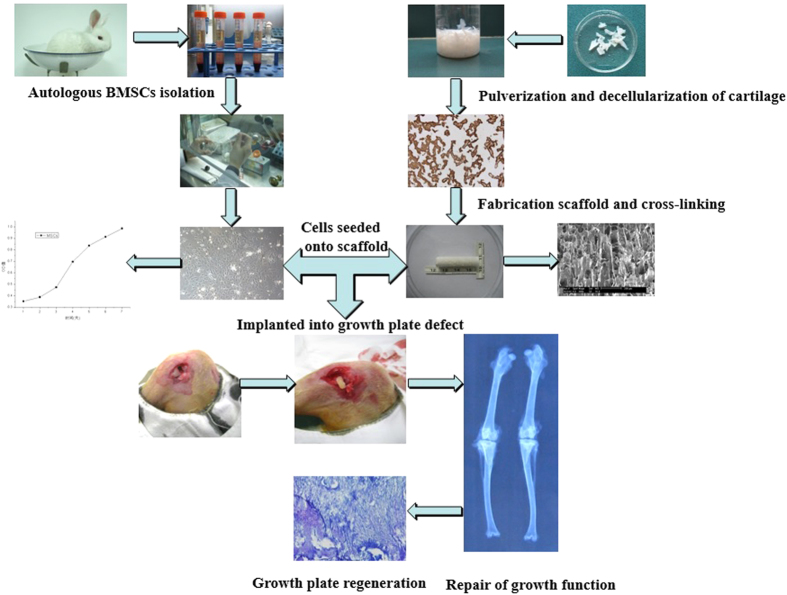
A schematic illustration of the experimental design of ECM oritented extracellular matrix scaffold for growth plate repair.

**Table 1:** Descriptive analysis of angular deformity and length discrepancy in different groups of rabbit tibia.

	Group	Times after operation				
		4 weeks	8 weeks	12 weeks	16 weeks	20 weeks
Angular deformity (°)	Group A	4.8 ± 1.33	9.4 ± 2.33	13.6 ± 3.32	14.2 ± 2.93	15.9 ± 3.83
	Group B	9.1 ± 1.32	13.9 ± 4.26^#^	23.6 ± 3.56^#^	26.8 ± 3.68^#^	27.1 ± 5.23^#^
	Group C	17.1 ± 4.50^†,*^	20.9 ± 3.12^†,*^	27.3 ± 6.24^*^	31.6 ± 3.58^*^	32.1 ± 2.85^*^
Length discrepancy (mm)	Group A	3.7 ± 2.11	5.4 ± 2.42	7.6 ± 2.87	8.1 ± 1.85	8.6 ± 1.69
	Group B	4.4 ± 1.69	7.2 ± 2.36	10.3 ± 2.14^#^	13.4 ± 2.54^#^	14.9 ± 3.09^#^
	Group C	7.8 ± 2.86^†,*^	10.8 ± 3.94^†,*^	12.8 ± 3.37^*^	15.3 ± 3.33^*^	17.1 ± 4.84^*^

^#^Means significant different between Group A and Group B (P < 0.001); ^*^means significant different between Group A and Group C (P < 0.001); ^†^means significant different between Group B and Group C (P < 0.001).
